# Nucleus incertus provides eye velocity and position signals to the vestibulo-ocular cerebellum: a new perspective of the brainstem–cerebellum–hippocampus network

**DOI:** 10.3389/fnsys.2023.1180627

**Published:** 2023-05-25

**Authors:** Guy Cheron, Laurence Ris, Ana Maria Cebolla

**Affiliations:** ^1^Laboratory of Neurophysiology and Movement Biomechanics, Université Libre de Bruxelles, Brussels, Belgium; ^2^ULB Neuroscience Institute, Université Libre de Bruxelles, Brussels, Belgium; ^3^Laboratory of Neuroscience, Université de Mons, Mons, Belgium; ^4^UMONS Research Institute for Health and Technology, Université de Mons, Mons, Belgium

**Keywords:** nucleus incertus, hippocampus, cerebellum, flocculus, vestibulo-ocular reflex, navigation

## Abstract

The network formed by the brainstem, cerebellum, and hippocampus occupies a central position to achieve navigation. Multiple physiological functions are implicated in this complex behavior. Among these, control of the eye–head and body movements is crucial. The gaze-holding system realized by the brainstem oculomotor neural integrator (ONI) situated in the nucleus prepositus hypoglossi and fine-tuned by the contribution of different regions of the cerebellum assumes the stability of the image on the fovea. This function helps in the recognition of environmental targets and defining appropriate navigational pathways further elaborated by the entorhinal cortex and hippocampus. In this context, an enigmatic brainstem area situated in front of the ONI, the nucleus incertus (NIC), is implicated in the dynamics of brainstem–hippocampus theta oscillation and contains a group of neurons projecting to the cerebellum. These neurons are characterized by burst tonic behavior similar to the burst tonic neurons in the ONI that convey eye velocity-position signals to the cerebellar flocculus. Faced with these forgotten cerebellar projections of the NIC, the present perspective discusses the possibility that, in addition to the already described pathways linking the cerebellum and the hippocampus via the medial septum, these NIC signals related to the vestibulo-ocular reflex and gaze holding could participate in the hippocampal control of navigation.

## Introduction

The cerebellum is involved in the long-range regulatory networks acting on different cortical regions to produce outcomes as predicted and, thus, avoiding sensorimotor and mental dysmetria (Schmahmann, 1991; Chaisanguanthum et al., 2014; Apps et al., 2018; Medina, 2019; Schmahmann et al., 2019). Numerous network models have been proposed and benefited from the exponential development during the last decade of deep learning computing technologies (Richards et al., 2019). The recently published study about cerebro-cerebellar network learning through feedback decoupling described by Boven et al. (2023) is a representative model that could explain how different lesions of the olivo-cerebellar circuit impact the learning ability of the whole brain. In this context, some brainstem areas devoted to crucial physiological functions, such as breathing (Feldman et al., 2003) and gaze holding (Tarnutzer et al., 2015), have feedback and feedforward connections with the cerebellum to reach an optimal behavioral state. Cerebellar integrity is crucial for optimal oculomotricity, maintaining the images of objects and environmental targets on the fovea. For this purpose, these cerebellar regions fine-tune the different subtypes of eye movements (Kheradmand and Zee, 2011): the flocculus/paraflocculus is involved in gaze holding, sustained pursuit, and vestibular reflex (VOR); the nodulus/ventral uvula contributes to the VOR; and the dorsal vermis and fastigial nucleus contribute to the initiation of saccades and pursuit. Both of these cerebellar regions are connected to the oculomotor neuronal integrator (ONI) location on both sides of the brainstem in the nucleus prepositus hypoglossi (PH) (Cheron et al., 1986a,b; Cannon and Robinson, 1987; Cheron and Godaux, 1987; Kaneko, 1997). Interestingly, in front of the ONI, the nucleus incertus (NIC) including the superficial part of the dorsomedial pontine reticular formation in and around the medial longitudinal bundle was implicated in oculomotricity (Cheron et al., 1995; Nakamagoe et al., 2000). In the present perspective, we propose revisiting a neglected but important grouping of NIC neurons that project to the cerebellum.

### Involvement of the NIC in arousal and hippocampal functions

In a recent review, Gil-Miravet et al. (2021) synthesized the important functions of the NIC, relaying neural signals about stress and arousal state to higher networks related to memory consolidation and retrieval. This review revisited the recent development of molecular, physiological, and behavioral evidence of the way in which this pontine nucleus exerts a widespread ascending modulation on emotional and cognitive process-related regions, among which are the medial septum (MS), the diagonal band of Broca, the prefrontal cortex, hippocampus (HPC), basal forebrain, and amygdala. However, nothing was mentioned about the NIC-cerebellar input. The NIC was indicated to have a major modulatory influence through at least two major processes. The first process consists of a fast regulatory action exerted by the GABAergic NIC neurons on the somatostatin-positive interneurons (SOMs) of the HPC involved in tonic inhibition of pyramidal cells. This disinhibition exerted by the NIC on the pyramidal cells of the HPC, which is responsible for formation of new contextual memories, is reinforced by the conjunction of another inhibitory action exerted by the NIC on the MS, which is known to exert strong excitatory input on the SOM. In parallel, a glutamatergic projection of the NIC (Cervera-Ferri et al., 2012) may participate in the modulation of the GABAergic, glutamatergic, and cholinergic neurons of the MS. These complex interactions between the NIC and the MS were revealed by an optogenetic study of the neuromedin B (NMB) neurons of the NIC providing both GABAergic and glutamatergic projections, which were differently involved in arousal processing, locomotor speed, and hippocampal theta oscillations in mouse (Lu et al., 2020). In addition, it is important to take into account the reciprocal input of GABAergic and glutamatergic MS neurons on the NIC and those from numerous regions of the brain (raphe regions, mammillary body, zona incerta, lateral habenula, and prefrontal cortex), which plays a pivotal role in the acquisition of contextual memories (Szonyi et al., 2019).

The second process concerns the role of the NIC in driving the modulation of the HPC theta rhythm (4–12 Hz). In accordance with NIC electrical stimulation inducing theta oscillations in the HPC (Nuñez et al., 2006), Cervera-Ferri et al. (2011) demonstrated that local field potential in the NIC synchronized with the HPC theta wave. In the same vein, a subgroup of NIC neurons presented coupled theta firing with the HPC theta oscillations (Martínez-Bellver et al., 2015). The same group demonstrated, by Granger causality analysis (Martínez-Bellver et al., 2017), a directional flow of synchronization between the theta oscillations of the NIC neurons and the HPC theta oscillations. Resetting of this latter oscillation by the NIC electrical stimulation has also been reported (Martínez-Bellver et al., 2017), demonstrating that the NIC plays a crucial role in the generation and modulation of the HPC theta rhythm.

How the inhibitory action exerted by the NIC on the MS and the disinhibition exerted by the NIC on the HPC pyramidal cells may affect the theta phase precession phenomenon remains largely unknown. However, Royer et al. (2012) demonstrated that optogenetic silencing of the SOM and PV interneurons produces different effects on the pyramidal cell behavior; namely, silencing of the SOM interneurons increased the burst firing without shifting the theta phase, whereas silencing of the PV interneurons had no outcome on burst firing, but significantly shifted the spikes' theta phase. More recently, Trenk et al. (2022) have identified two different types of NIC neurons: the theta phase-independent and theta bursting neurons. The former exert a permissive function on the MS, while the latter are implicated in the synchronization of the theta HPC oscillations. The relationship between the evolution of the phase of the theta oscillations and the spiking of a place cell when coding for a specific position was recognized as the precession phenomenon and considered one of the fundamental mechanisms for understanding the place cell and theta oscillation behavior (O'Keefe and Recce, 1993). Because it was demonstrated that different subcortical regions presenting theta oscillations were able to modulate the theta-HPC, it is possible but not yet proven, that the theta oscillations of the NIC could also modulate the entorhinal cortex. The NIC signals could participate in the oscillatory interference model (O'Keefe and Burgess, 2005) in which multiple frequency band oscillations reach different dendrites of the same neuron determining the grid cell's behavior (Hafting et al., 2005) and converge to the emergence of the place cells in the HPC (Burgess et al., 2007; Burgess, 2008). The importance of theta oscillations for episodic and associative memory, movement, and navigation in both rodents and humans was recently reinforced (Bohbot et al., 2017; Bush et al., 2017; Goyal et al., 2020; Kota et al., 2020; Vivekananda et al., 2021). This motivates new exploration of the subcortical and cortical organization of the theta oscillations considered to be a timing template encoding the sequence of movement and planned navigational trajectories (Buzsáki, 2005; Nuñez et al., 2006; Nuñez and Buño, 2021). Recently, Rondi-Reig et al. (2022) placed the NIC into a privileged position between the fastigial nucleus on one side and the MS linked to the diagonal band of Broca and the HPC on the other side. This functional axis is reinforced by other parallel pathways, involving the fastigial nucleus to the supramammillary nucleus and median raphe nucleus. This latter nucleus is also connected with the dentate nucleus and HPC. This ensemble of structures is considered a “theta-pathway”, assuming a strong functional link between the cerebellum and HPC.

### Forgotten role of the NIC

**Figure 1:**
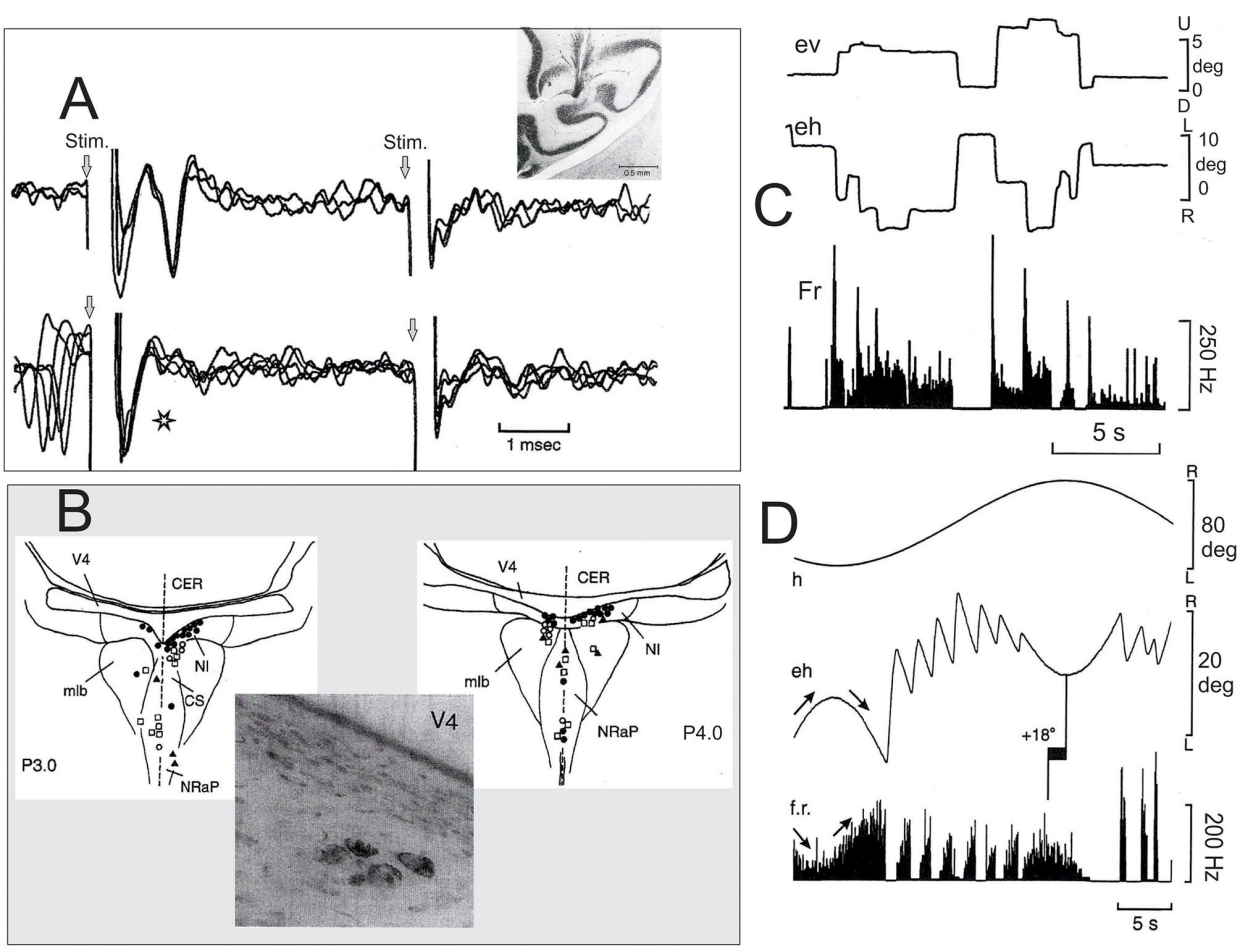
Characterization of neurons of the nucleus incertus (NIC) projecting to the cerebellar flocculus. **(A)** Top, superimposition of three recording traces showing the antidromic activation of a burst tonic (BT) neuron of the NIC antidromically activated by the contralateral flocculus (see the histological section showing the placement of the stimulating electrode in the middle zone of one flocculus) and the lack of activation of the same unit from the ipsilateral flocculus. Bottom, collision testing. The star indicates an absence of the antidromic spike when it collided with the spontaneous spike. **(B)** Localization of the floccular projecting neurons in the pontine reticular formation (PRF) at the P3 (left) and P4 (right). The photograph illustrates a cluster of NIC neurons (P.4, Nissl counterstain) retrogradely labeled after HRP injection in the middle zone of the contralateral flocculus. **(C)** The behavior of one representative BT neuron of the NIC during spontaneous eye movements. **(D)** The behavior of the same NIC neuron during the horizontal vestibulo-ocular reflex. Note that, in this case, the phase lead of the firing rate modulation is 18 deg for eye position. Abbreviation: mlb, medial longitudinal bundle; CS, central superior nucleus of the raphe; NRaP, nucleus raphe pontis; CER, cerebellum; V4, fourth ventricle; ev, vertical eye position; eh, horizontal eye position; f.r., firing rate; h, head position (Adapted from Cheron et al., 1995).

In this general context of movement control, two main elements are lacking in the majority of studies devoted to the NIC; the first is its role in oculomotricity, and the second is its relation to the cerebellum. Cheron et al. (1995) explored pontine reticular formation in the awake cat to identify the neurons that are antidromically activated by the horizontal zone of the flocculus. These electrophysiological explorations were motivated by a previous study in the anesthetized cat (Nakao et al., 1980) that showed antidromic activation of neurons located in and around the medial longitudinal bundle, in the nucleus raphe pontis (NRaP), and the nucleus reticularis tegmenti pontis in response to electrical stimulation of the flocculus. Based on this anesthetized preparation, a new population of neurons inside the NIC was found in the awake cat (Cheron et al., 1995). The projection of these neurons to the flocculus was certified by collision testing between the spontaneous spikes and antidromic potentials (Figure 1A). Thirty-eight NIC neurons were activated from the contralateral flocculus at a latency of 1.32 ± 0.45 ms, and 17 NIC neurons were activated from the ipsilateral flocculus at a latency of 1.16 ± 0.34 ms, indicating a rapid transmission of the NIC signals. The firing behavior of these NIC cerebellar projecting neurons was well characterized. First, during eye saccades, the NIC neurons stopped to fire when the eyes moved toward the recording site and produced a burst at a high frequency (~300 Hz) when the eyes moved in the opposite direction. After the burst, during intersaccadic fixation, they presented tonic firing that was linearly related to the eye position, with increased firing for the eccentric position toward the contralateral side. Second, during the vestibulo-ocular reflex (by sinusoidal rotation), these NIC neurons showed a type I sinusoidal modulation interrupted by bursts and pauses corresponding to the quick phases, directed away or toward the recording side, respectively (Figure 1B). Such burst tonic (BT) behavior was also described in other brainstem regions (medial vestibular and PH nuclei) projecting to the cerebellar flocculus (Cheron et al., 1996; Escudero et al., 1996).

### Function of the NIC in flocculus–oculomotor integrator relationships

**Figure 2:**
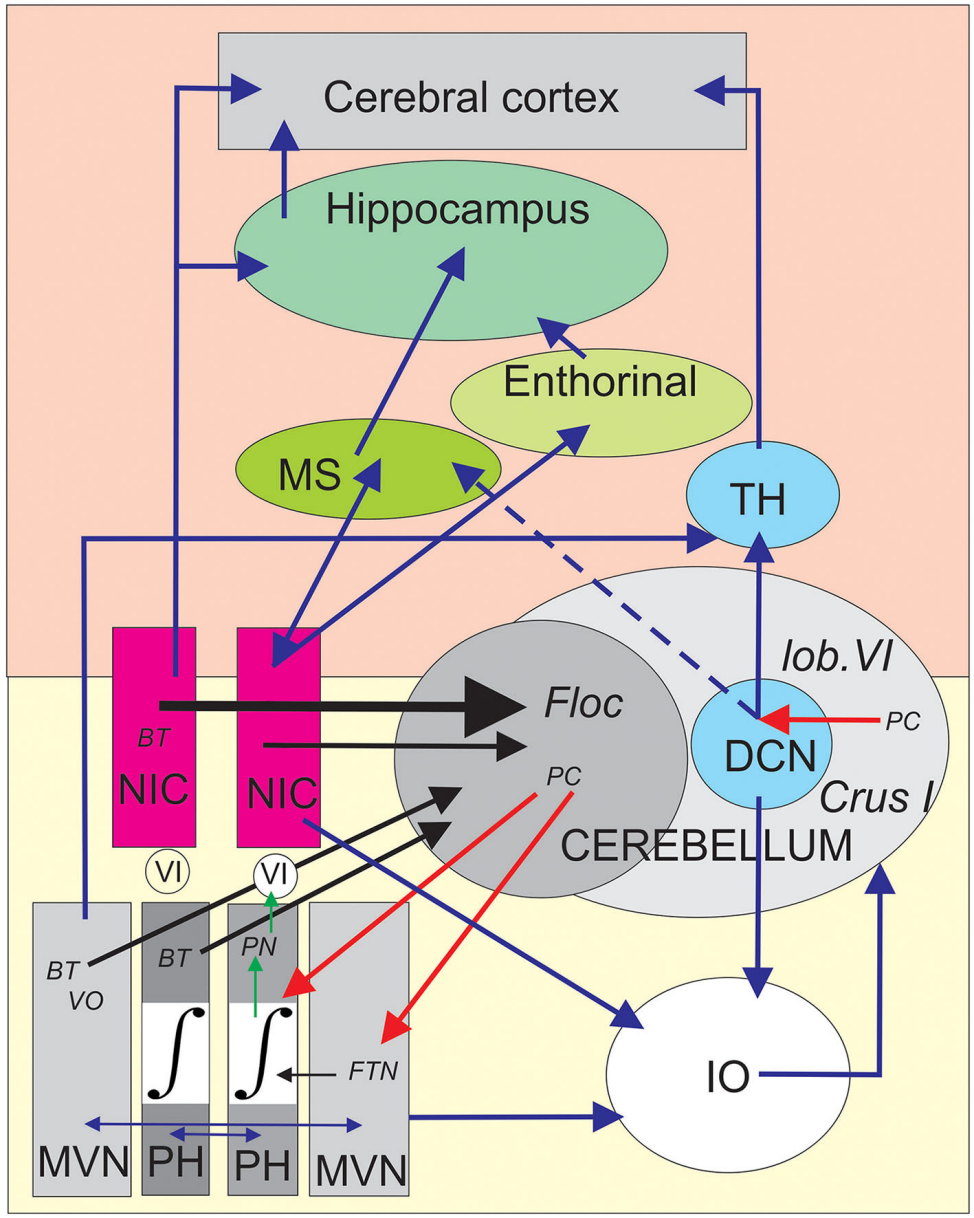
Schematic depiction of the central position of the nucleus incertus (NIC) into the proposed brainstem–cerebellum–hippocampus network. The yellow area (bottom) symbolizes the brainstem olivo-cerebellar circuit responsible for gaze-holding behavior involving the medial vestibular nuclei (MVN), the oculomotor neural integrator (∫) situated in the prepositus hypoglossi (PH) nuclei. The burst tonic (BT) neurons projecting to the cerebellar flocculus (Floc) were antidromically identified in the contralateral MVN, PH, and NIC and marked by black arrows. In addition, the vestibular input reaches the flocculus through the vestibular-only (VO) neurons of the contralateral MVN. The collicular connections to the excitatory and inhibitory burst neurons (EBN and IBN) are not illustrated for clarity. The Purkinje cells (PCs) of the flocculus provide inhibition on the floccular target neurons (FTNs), which are treated as the EBN and IBN velocity neurons by the oculomotor neural integrator to produce the final position neurons (PNs). These PNs project this signal to the motoneurons of the abducens nucleus (VI) of the same side. The vestibular and prepositus nuclei are reciprocally connected by commissural fibers assuming the vestibular balance and velocity storage (horizontal blue arrows). The vestibular-prepositus complex, the NIC, and the deep cerebellar nuclei (DCN) neurons are linked to the inferior olive (IO), producing a corollary discharge that allows the comparator function of the IO to the cerebellar network. The pink area (top) symbolizes a simplified version of the main elements of the general memory and navigation system connected to both the NIC region and the cerebellum. The NIC presents reciprocal connections with the medial septum (MS), and then to the hippocampus, where the place cells were founded. The NIC also projects to the entorhinal cortex, where the grid cells are located. Clusters of PCs in the lobule VI and Crus I projecting to the DCN which reach the hippocampus and the cortex from the thalamus (TH). The black arrows represent antidromically identified neuronal projections, the blue arrows represent anatomical connections, the red arrows represent the physiological inhibition of PCs, and the green arrows represent electrophysiologically identified projections.

Following the NIC floccular projections carrying BT oculo-vestibular signals, data from Nakamagoe et al. (2000) reinforced the idea that the brainstem neurons in and around the paramedian tract (PMT) region (including the NIC) constitute crucial sensory-motor input to the cerebellar flocculus. Interestingly, pharmacological inactivation (muscimol microinjection) of the PMT-NIC neurons results in a gaze-holding failure of both horizontal and vertical saccades (Figure 3 of Nakamagoe et al., 2000) and vestibular imbalance. Based on the fact that these pathological eye movements resemble (Robinson, 1974; Godaux and Vanderkelen, 1984) suppression of the oculomotor integrator situated in the PH (Cheron et al., 1986a,b; Cannon and Robinson, 1987; Cheron and Godaux, 1987; Kaneko, 1997), Nakamagoe et al. (2000) proposed that the neurons of the PMT region contribute to a brainstem–cerebellar network by forming a leaky cerebellar integrator. This cerebellar integrator participates in the final positional adjustment of the eyes (Robinson, 1989; Cheron et al., 1997). The strong eye velocity signal of the BT neurons of the NIC projecting to the flocculus (Figure 1C) could participate in the generation of the eye velocity command elaborated by the flocculus during the pursuit (Joshua and Lisberger, 2015). This velocity signal of the flocculus has been identified as one of the major elements of pursuit behavior initiation through the floccular target neuron (FTN) (Figure 2) situated in the vestibular nuclei (MVN) (Joshua et al., 2013). It also represents one of the velocity signals used by the common oculomotor integrator for positional control of the eye during pursuit. It could be possible that the velocity signal transmitted by the NIC to the flocculus participates in the gaze-holding system (Figure 2).

### Function of the NIC-cerebellar input in hippocampal learning and navigation

The eye movement NIC neurons has not been recorded during the classical testing used for emotion (e.g., contextual fear memory), navigational context (e.g., T-maze, place field, water maze), and locomotion (e.g., wheel running). However, considering that the pre-motoneurons of the paramedian pontine reticular formation take into account the head velocity (Cullen and Guitton, 1997), the comparison of the abducens motoneuron discharges during head-restrained and head-free movements demonstrated that the head-restrained dynamics remain applicable in head-free conditions (Cullen et al., 2000). It is, thus, highly probable that regardless of the behavioral context implicating head and eye movements, the same BT behavior would be present and transmitted to the cerebellar flocculus. With their high sensitivity for both eye velocity and eye position, these NIC neurons could provide an efference copy of head–eye movement commands to the flocculus. Combined with other types of cerebellar input, the NIC signals would contribute to one of the main functions of the vestibulo-cerebellum, to combine vestibular, visual, and eye–head-related movements into an internal dynamic model (Laurens and Angelaki, 2020). This neuronal operation may contribute to producing forward signals that are behaviorally relevant (Delgado-García, 2001; Kim et al., 2019). In addition to a well-known role in motor learning, the cerebellum plays a role in cognitive processes, such as those involved in spatial navigation. This latter represents a goal-directed behavior requiring the formation of an HPC spatial map (Petrosini et al., 1996; Mandolesi et al., 2001; Burguière et al., 2005). Another interesting pathway concerning both the PH and NIC is provided by the fastigial projections. Fujita et al. (2020) identified two different regions of the fastigial nucleus (modules F2 and F4) specifically dedicated to orienting head–eye movements in connection with the PH and to vigilance/activation of the HPC in connection with the NIC. A goal-oriented navigation task is a perfect example of a basic behavioral task involving a large part of the brain. It implies multimodal declarative learning and procedural learning, via the HPC and basal ganglia, respectively, as well as assimilation of task rules and sensorimotor adaptation via the prefrontal cortex and cerebellum, respectively. Selective disruption of cerebellar protein kinase C at parallel fiber-Purkinje cell synapses in transgenic L7PKCI mice has been shown to induce important deficits in the self-motion processing used in hippocampal navigation performance (Rochefort et al., 2011; Lefort et al., 2019). In this context, Watson et al. (2019), using a retrograde transneuronal tracer, identified cerebellar inputs transmitted from the deep cerebellar nuclei to the dorsal HPC that came from a disparate cerebellar cluster in the lobule VI, Crus I, and paraflocculus. However, this study demonstrated that the connection between the deep cerebellar nuclei and HPC is not direct (at least disynaptic), implicating single-relay in the MS and supramammillary nucleus. These authors highlighted the fact that the cerebellum dynamically interacts with these relay stations through theta-alpha oscillation (6–12 Hz) to potentially modulate those involved in the theta phase precession phenomenon observed in the activity of hippocampal place cells (O'Keefe and Recce, 1993).

## Discussion

Until recently, the cerebellar input signals of the NIC were only described for the cerebellar flocculus (Cheron et al., 1995). The target region of Purkinje cell inhibition output in the vestibular complex, which is considered to be a deep cerebellar nucleus, must be taken into account as a potential contributor to hippocampal or cortical functions. Although whether the same NIC neurons project to the cerebellum and HPC is unknown, we speculate that the BT oculomotor signals of the NIC contribute to some of the HPC functions. This latter option is supported by numerous pieces of evidence (de Waele et al., 2001; Lopez and Blanke, 2011; Kirsch et al., 2016) showing that some neurons of the vestibular complex are connected via the thalamic relay to the HPC and the parieto-insular vestibular cortex. The functional connections between the NIC, flocculus, and ONI could be viewed as a complementary contribution of the gaze-holding system to the other cerebellar interconnections through the fastigial nucleus, medial vestibular nucleus, and PH implicated in head motion control during navigation (Fujita et al., 2020; Rondi-Reig et al., 2022).

Thus, we conclude this perspective view with the expectation that the vestibulo-ocular function of the NIC and NIC-related cerebellar output toward the HPC and cerebral cortex should be considered a significant part of the neural network of navigation. This hypothesis will necessarily have to be tested by electrophysiological NIC neuron recordings to confirm whether the same or different neurons convey vestibulo-ocular signals to the cerebellum, HPC, and cerebral cortex. Concretely, it would be interesting to check in a first protocol whether NIC neurons projecting to the cerebellum also project to the MS and HPC and whether they exhibit theta oscillatory activity. To do so, stimulating electrodes should be placed in the cerebellum, MS, and HPC to identify whether NIC neurons recorded during the behavioral task are commonly antidromically activated or whether they are activated by different neurons from different structures. In a more complex protocol, the same electrophysiological identification could be made on optogenetic preparations.

## Data availability statement

The original contributions presented in the study are included in the article/supplementary material, further inquiries can be directed to the corresponding author.

## Author contributions

All authors listed have made a substantial, direct, and intellectual contribution to the work and approved it for publication.
